# The Late Surgeon-Major Parke

**Published:** 1893-09-16

**Authors:** 


### The Late Surgeon-Major Parke.

In the death of Surgeon-Major Thomas Heazle Parke, a chivalrous gentleman and a true knight of medicine, has passed away from his professional brethren and the world. Dr. Parke has only been in practice fifteen years, and he could barely have been forty years of age. He was on a visit to the Duke of St. Albans at Altna-Craig, and died suddenly on Sunday night. It is impossible to write without emotion of the unexpected and untimely death of one so young, so honourably known, and so universally beloved. Dr. Parke was above all things an "African doctor." Africa has claimed of the Western world some of the bravest, the most patient, the most strenuous, and the most richly endowed of all its children. Dr. Parke was great even among the greatest of those unconsciously heroic personalities who have revived for us all the chivalrous romance of early historic ages, in African story. Writing four years ago at Kavalli, Mr. H. M. Stanley said of Parke: "This expedition (the Emin Relief Expedition) possesses the rarest doctor in the world. No country in the world can produce his equal in my opinion. There may be many more learned perhaps, more skilful, older or younger as the case may be; but the best of them have something to learn from our doctor. He is such a combination of sweetness and simplicity; so unostentatious, so genuinely unobtrusive. We are all bound to him with cords of love. We have seen him do so much out of pure love for his 'cases' that human nature becomes ennobled by this gem. He is tenderness itself. He has saved many lives by his devoted nursing. . . . At Abu Klea our doctor was great; the wounded had cause to bless him; on the green sward of Kavalli, daily ministering to those suffering blacks, unknowing and unheeding whether any regarded him, he was greater still." Of honours from men and societies who know how to select those who are worthy of honour, Dr. Parke had many; and of enduring work for his profession he had done much. He was Honorary D.C.L. of Durham, he had received from the Sultan the Order of the Medjidieh and the Brilliant Star of Zanzibar. He was a Gold Medallist of the British Medical Association, and a Fellow of the Royal Geographical Society, as well as of many other similar societies. Dr. Parke was the author of "Experiences in Equatorial Africa," a "Report to the War Office on the Cholera Outbreak in Egypt," and of various other publications and contributions to the medical and scientific journals. Quite recently he had published a "Guide to Health in Africa," of which so competent an authority as Mr. H. M. Stanley declared "few men were so well qualified as he to instruct the missionary, the traveller, the merchant, the miner, and the soldier in the secrets of African diseases."

All those Universities and societies which selected Dr. Parke for honour whilst he was yet young, and could value such honours, will feel glad that they did not delay their recognition of the rare merit of so rare a man. The Governments of our day, both Conservative and Liberal, will feel a pang—if Governments can feel pangs—that not one sign of recognition, great or small, was bestowed upon him by the State from first to last. As loyal members of a great citizenship, we cannot but feel painful emotion that a man who was so universally honoured and beloved should have been passed over by the statesmen of the day simply because his work happened to be that of life-saving and not of fighting. But, indeed, the lives of such men as Parke often carry our conquests farther into the recesses of uncivilised lands than the rifles and bayonets of our soldiers. The late Government and the present one both erred in their cold indifference to medicine as it showed itself in Dr. Parke. Let us hope that his untimely death will bring a tinge of shame to some faces which, now that it is too late, would perhaps willingly do him honour.

